# Some Remarks on Extracting, from an Orthodontist’s Point of View

**Published:** 1902-01-15

**Authors:** Milton T. Watson


### DENTAL REGISTER.

Vol. LVI. January 15, 1902. No. 1.

### SOME REMARKS ON EXTRACTING, FROM AN ORTHODONTIST'S POINT OF VIEW.

BY MILTON T. WATSON, D.D.S.

Read before the Washtenow County Dental Society, at Ann Arbor, Mich., November 10, 1901.

Slowly indeed has orthodontia emerged from its dark and uncertain past until to-day it is as much a science as is the practice of surgery, and is already demanding in its practice the exclusive time and attention of men. One of the best evidences of this evolution is the fact that teachers are now beginning to devote time to the consideration of both the normal and pathological conditions of the parts involved, and are thus enabled to make a careful and accurate diagnosis and prognosis before beginning treatment, instead beginning it as of old without any knowledge of just what the outcome was going to be, as was always the case when the consideration of this subject covered only the mechanical phase, or that part which had to do with the designing and construction of innumerable appliances to be used in the attempt at correcting malocclusions, the true character of which the student, and often the teacher had not the slightest conception.

Little wonder then that in the past a large majority of the operations attempted have failed utterly to accomplish the movement desired, or that when seemingly, the movement was accomplished that the patient was often worse off than in the beginning; for while possibly the teeth had been arranged symetrically, this change had come about at the expense of badly distorted facial lines. These dire results have stimulated men who are interested in orthodontia to make a more careful study of the human face, and to-day the practice of orthodontia demands that a man consider, equally as carefully, the facial requirements as he does the occlusal requirements.

In our efforts at higher education, I beg to prophesy that the day is sure to come when the study of fine art will occupy a prominent place in the dental curriculum. In order to successfully execute his work, the orthodontist and the dentist, who are constantly remodeling the human face, should possess a keen esthetic taste, and in no way can this be better developed than by at least a fundamental study of the fine arts, I would not be understood as advocating a lower standard of mechanical ability, but rather as urging the necessity for a study that will sharpen our sense of the harmony of things in general and particularly of the facial lines, and that will forever stamp a man as a criminal, who will extract the roots of anterior teeth and attempt to replace the loss with bridge work, when it would be possible by any means whatever to restore these roots to a healthful condition, and, by this means preserve the normal contour of the face, instead of sending the patient through life with that sunken expression that ever results from the loss of the roots of these teeth, provided they were approximately in their normal position.

I have known this operation to be performed many times when it could easily have been avoided, and by men too, who would not intentionally injure a patient. They had never received any instruction as to the effect upon the face, of extracting these teeth, and were not sufficiently keen observers to have discovered it for themselves. I have also known men to advise the extraction of perfectly sound upper incisors because they were in a "twisted and crowded" condition, or in one case simply because they were in a position of infra occlusion, assuring the patient that beautifully even teeth could be put in the place of the irregular ones extracted, and they were apparently honest in the belief that such was really possible, never dreaming that they were deforming their patient irreparably—and all this because the facial aspect of dentistry receives too little attention.

It seems hardly necessary to speak of the lack of refined taste which tolerates such unsightly things as gold crowns on anterior teeth, and the insertion of "white china teeth" in the mouths of elderly patients, yet the fact remains, that, go where we will in this country, which is supposed to be almost burdened with expert dentists (the fact is, expert mechanics) such glaring work is ever in evidence to convict us as a profession, of extremely bad taste.

Real esthetic taste, like fine mechanical ability, is natural to some men, but the great majority of us need to cultivate it quite as much as we need to cultivate the mechanical. The artist or the sculptor would be an utter failure without a well-developed sense of balance and harmony; and what applies so forcibly to the man who chisles a marble face, has a vastly greater importance to the man who is moulding the face of a human being, whether it be through the influence exerted by the insertion of artificial teeth on plates or bridges, or through the immense possibilities in orthodontia.

In this connection and because of its marked influence upon the face, I can not refrain from making an earnest appeal to you to stop that practice of extracting the first molars in the hope of relieving a crowded condition of the front teeth. I am unalterably convinced that we add to malocclusion, though in a different form perhaps, oftener than we relieve it by extracting these teeth—however there are undoubtedly some cases under the influence of strong lip pressure in which the front teeth do drop back, but in so doing, the already diminished width of the jaw is made a permanent deformity, and the support that one tooth affords another in the normal jaw is usually destroyed.

The injury to the masticating capacity alone, of the second and third molars, in my judgment, stamps this as an operation extremely injurious to their most important office, and the marring of the facial appearance is usually an irreparable condition, for unless the lips are unusually thick, the development of the face in the region of the mouth will be decidedly deficient, in many cases amounting to a markedly depressed appearance. The argument is used that the face does not change contour because the second molar comes forward and takes the place of the first—all right—what, then, becomes of the second molar space? The answer comes that the third molar occupies this—again all right, but now what becomes of the space the third molar would occupy in a normal occlusion? The forceful answer must come that little if any of it remains, showing beyond doubt that the jaws have failed to develop in an antero-posterior direction to almost the mesio-distal diameter of a molar tooth and if it be true that the jaws are that much shorter than they should be, who, with any knowledge of the facial requirements, can deny that this almost universally practiced operation is largely responsible for a goodly number of the deformed jaws, and unbalanced faces with which we come in contact almost daily? (It is of course understood that I refer to extraction during childhood).

The theory of this operation is again defective, for if the second molar always migrated forward, completely occupying the place formerly occupied by the first, it would be impossible for the bicuspids and the cuspid to settle back and relieve the crowded condition of the incisors, and if it does not always come forward completely occupying the space formerly occupied by the first molar, and the teeth in front of it do recede, then you are bound to admit that the facial appearance, in the region of the mouth must be depressed by such an operation, not only to the extent to which they would have been carried forward by the normal development of the jaw, had all the teeth remained in place.

The men who advocate the extraction of these teeth sometimes tell us that the third molar erupts a much stronger tooth as a result of such an operation, and that thus the defective first molar is replaced by the sound second molar, and the third molar which under ordinary circumstances would be lost early, remains in place a strong useful tooth, and instead of having one of the two molars only, which most people retain for any great length of time, a defective tooth, they are both sound teeth. This sounds well—but the real facts are simply that the surrounding conditions are more favorable for its complete eruption and proper cleansing. In that most men who have adopted this operation do not do the extracting before eleven years of age, at which time the crown of the third molar is usually completely calcified (Bromell) makes it evident that the physical character of the tooth can not be affected favorably or otherwise.

To those who are performing this operation because they honestly believe they are doing their patients a good service, (I have nothing to say to the man who does it because it is an easy way out of a difficulty), I earnestly beg you to study the results, not from the mouth alone, for this does not show the lingual occlusion, but from carefully made and articulated models, always making comparisons from equally carefully made and articulated models of a normal occlusion, and have uppermost in your mind the thought that mastication is the really important work of the teeth—the esthetic phase must of course be studied from the face. After a careful study of this kind I feel confident you will discard this, to my mind, really destructive operation, as another of the methods tried and oftener than otherwise, found wanting, for the very evident lessened usefulness of the teeth will usually be sufficient to convince you even without considering the esthetic conditions.

Do not misunderstand me—I am aware that there are many cases presented to the general practitioner where it is impossible to save these teeth, and in such cases we must of course submit to the inevitable—but let it be *only* to the inevitable.

The orthodontist is rarely, if ever, confronted by more perplexing obstacles than when little patients are referred to him after the removal of these molars. He hesitates to send the patient back to the family dentist with the request that the lost teeth be replaced, especially after the family dentist has assured the parents that there was no other way to provide room for all the teeth, however, notwithstanding the embarrasment of the position, it is often a physical impossibility to bring about results even approximating the normal unless we make good the loss of these valuable organs of mastication and essential means to an end in the development of the face.

The position is an awkward one when cases are referred even after the extraction of a bicuspid, but we can many times manage such cases in a way that will greatly improve the existing conditions, both in respect to the occlusion and the esthetic requirements notwithstanding the loss, though on some occasions I have found it absolutely necessary to have them replaced in order to bring about the normal facial expression.

I am completely at a loss to understand, how, in the treatment of malocclusion, any man can ever extract an incisor tooth, after he has made a careful study of the importance each tooth bears not only to those with which it comes in immediate contact, but to the masticatory apparatus as a whole. The arch is bound to be made narrower by the width of the tooth removed, and in that it is an invariable law in the arrangement of the teeth that there must be harmony in the size of the arches—this narrowing of one jaw will surely be compensated for by some of the teeth in the other jaw assuming a position of malocclusion.

A little careful observation will convince any thoughtful student that these contracted arches go hand in hand with conditions which it should be our aim to avoid rather than induce, and to correct rather than perpetuate when we find they already exist. I refer to such baneful effects of malocclusion as interference with normal nasal respiration, impaired masticating capacity, and ability to enunciate distinctly, as well as the "pinched expression" of a face that is moulded over a contracted bony framework.

