# Correction

**Published:** 1902-04-15

**Authors:** 


*Correction:*—On page 5 of the January issue the following sentence should be inserted following the words "not only," in the twentieth line: "to the extent to which the front teeth have receded, but also." This is the article on extracting from an orthodontist's view, by Dr. Watson.

